# Androgen-induced cerebral venous sinus thrombosis in a young body builder: case report

**DOI:** 10.1186/1471-2377-4-22

**Published:** 2004-12-03

**Authors:** Mohammad Ali Sahraian, Mahmood Mottamedi, Amir Reza Azimi, Babak Moghimi

**Affiliations:** 1 Department of Neurology, Sina Hospital, Tehran University of Medical Sciences, Hassan Abad Square, Imam Khomini Street, Tehran, Iran

## Abstract

**Background:**

Cerebral venous sinus thrombosis is an infrequent disease with a variety of causes. Pregnancy, puerperium, contraceptive pills and intracranial infections are the most common causes. The patient may present with headache, focal neurological deficits and seizures.

The clinical outcome is highly variable and treatment with heparin is advised.

**Case presentation:**

The patient is a 22 year old male who presented with headache, repeated vomiting and papilledema.

He was a bodybuilder doing exercise since 5 years ago, who had used nandrolone decaonoate 25 milligrams intramuscularly during the previous 5 months. Brain MRI and MRV showed superior sagital and transverse sinus thrombosis and extensive investigations did not reveal any known cause.

**Conclusions:**

We suggested that androgen was the predisposing factor in our patient. Androgens may increase coagulation factors or platelet activity and cause arterial or venous thrombosis.

As athletes may hide using androgens it should be considered as a predisposing factor for thrombotic events in such patients.

## Background

Cerebral venous sinus thrombosis (CVST) is a disease with a wide spectrum of non specific clinical signs and symptoms, including headache, focal neurological deficits, seizures and coma.

The clinical outcome is highly variable; patients may recover completely or may develop severe and lasting neurological deficits [1].

There are many causes for this disease but the most common predisposing factors are pregnancy, puerperium, contraceptive pills, coagulopathies and intracranial infections [2].

There are few reports of patients with CVST after androgen therapy. We present a young bodybuilder man who developed CVST with abusing androgens for increasing muscle mass.

## Case presentation

In May 2004 a 22 year old male was admitted to our department with chief complaints of headache and vomiting.

The patient was well till 10 days prior to admission that developed progressive, intense bitemporal headache exacerbated with bending.

The patient also had history of malaise, nausea and several episodes of vomiting from 3 days before admission. The only objective finding on physical examination was bilateral papilledema. The patient was a body builder doing exercise from 5 years ago who had used nandrolone decaonoate 25 mg once or twice a week during the last 5 months. He had injected 20 ampoules in this period.

Brain computed tomography without contrast was done for the patient which showed cord sign, emergency MR imaging including T1 – T2, weighted and MRV showed prominent superior sagital and transverse sinus thrombosis. The C.S.F opening pressure was 480 mm/H2o without any other abnormality. Heparin 80 IU/kg started as loading dose then continued 1000 IU/ hr for 10 days. On the 5^th ^day of treatment headache resolved and warfarin added to heparin.

Laboratory tests including antithrombin III activity, protein C, S factor V leiden, Plasma hemocystein and anticardiolipin were all within normal limits.

The patient was discharged in a good condition and was maintained on 6 months warfarin protcol.

## Discussion

There are few reports of CVST following androgen therapy [3], but there is just one reported case of CVST in androgen using young body builder [4]. The anabolic activity of testosterone and its derivatives is primarily manifested in its myotrophic actions which result in greater muscle mass and strength.

This has led to widespread use of androgenic anabolic steroids by athletes at all levels. Nandrolone decaonoate is a synthetic anabolic steroid. In focus on homeostasis system the most important factors under testosterone regulation are fibrinogen, Plasminogen activator inhibitor-1 (PAI – 1) and platelet aggregability.

The current data indicate that testosterone lowers fibrinogen and PAI – 1, however these anticoagulatory and profibrinolytic may be opposed by proaggregatory effects on platelets because high dosages of androgens were found to decrease cycloxygenase activity and thereby increase platelet functions [5].

Proaggregatory effect of testosterone and other synthetic androgens become more reliable theory for CVST, according to recent publication [6].

## Conclusions

This case report presents a patient with CVST following exogenous androgen usage with a mechanism which is not completely understood, but it may be related to platelet activation or an increase in coagulation factors. As androgen use may be frequent and hidden in athletes, it may be an underestimated cause of cerebral venous thrombosis in young adults and careful history should be taken in these groups of patients.

## Lists of abbreviations

CVST: cerebral venous sinus thrombosis

PAI-1: Plasminogen activator inhibitor-1

MRI: magnetic resonance imaging

MRV: magnetic resonance venography

## Competing interests

The author(s) declare that they have no competing interests.

## Authors' contributions

M.A.S: Admitting and treating the case, preparing the article.

M.M : Revising the article.

A.R.A : searching previous articles, preparing case presentation part.

B.M : searching previous articles, preparing background.

**Figure 1:**
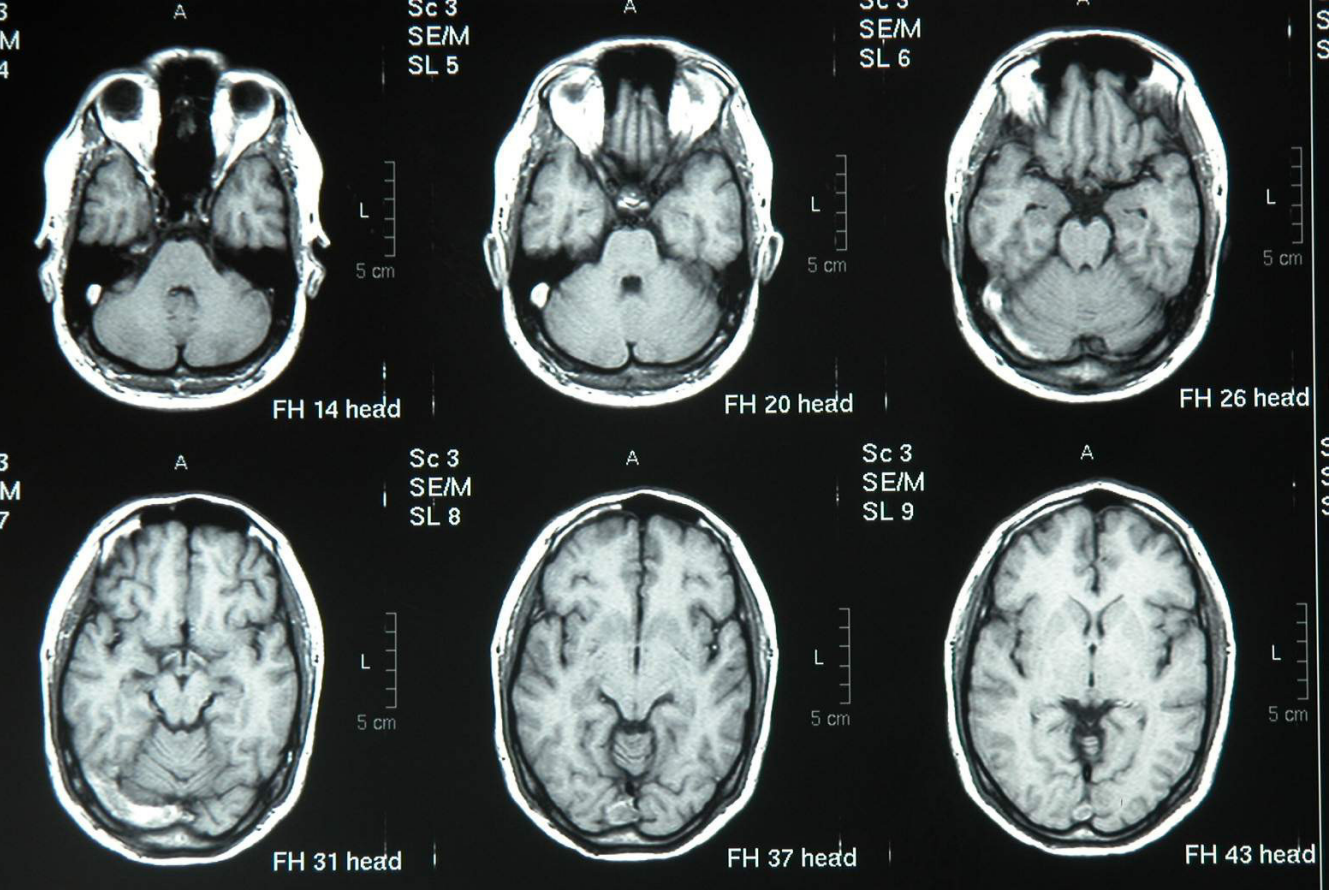
T1 weighted horizontal nonGd Images show transverse sinus thrombosis.

**Figure 2:**
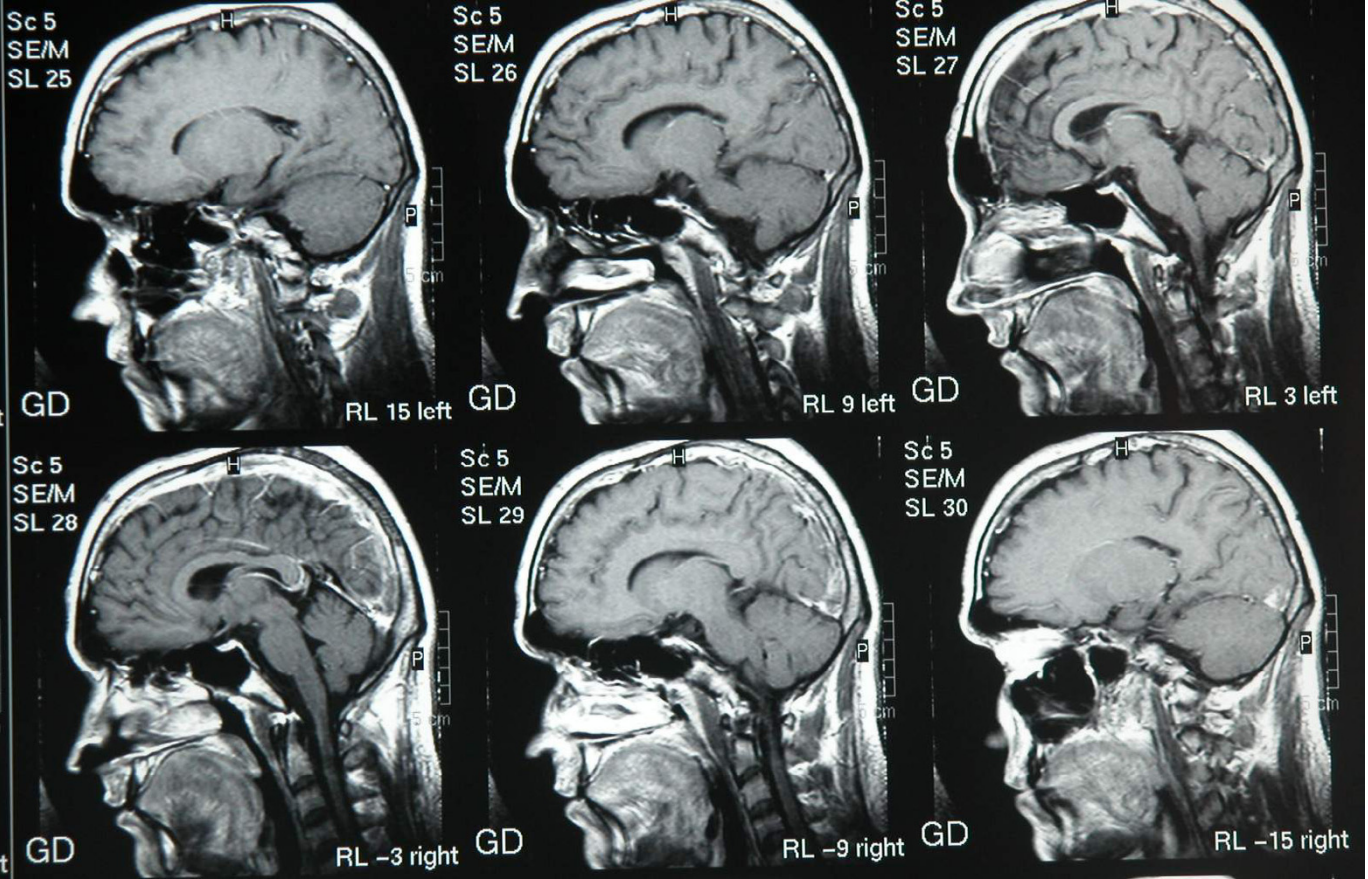
T1 weighted sagital Images with Gd show thrombosis in sagital sinus.

**Figure 3:**
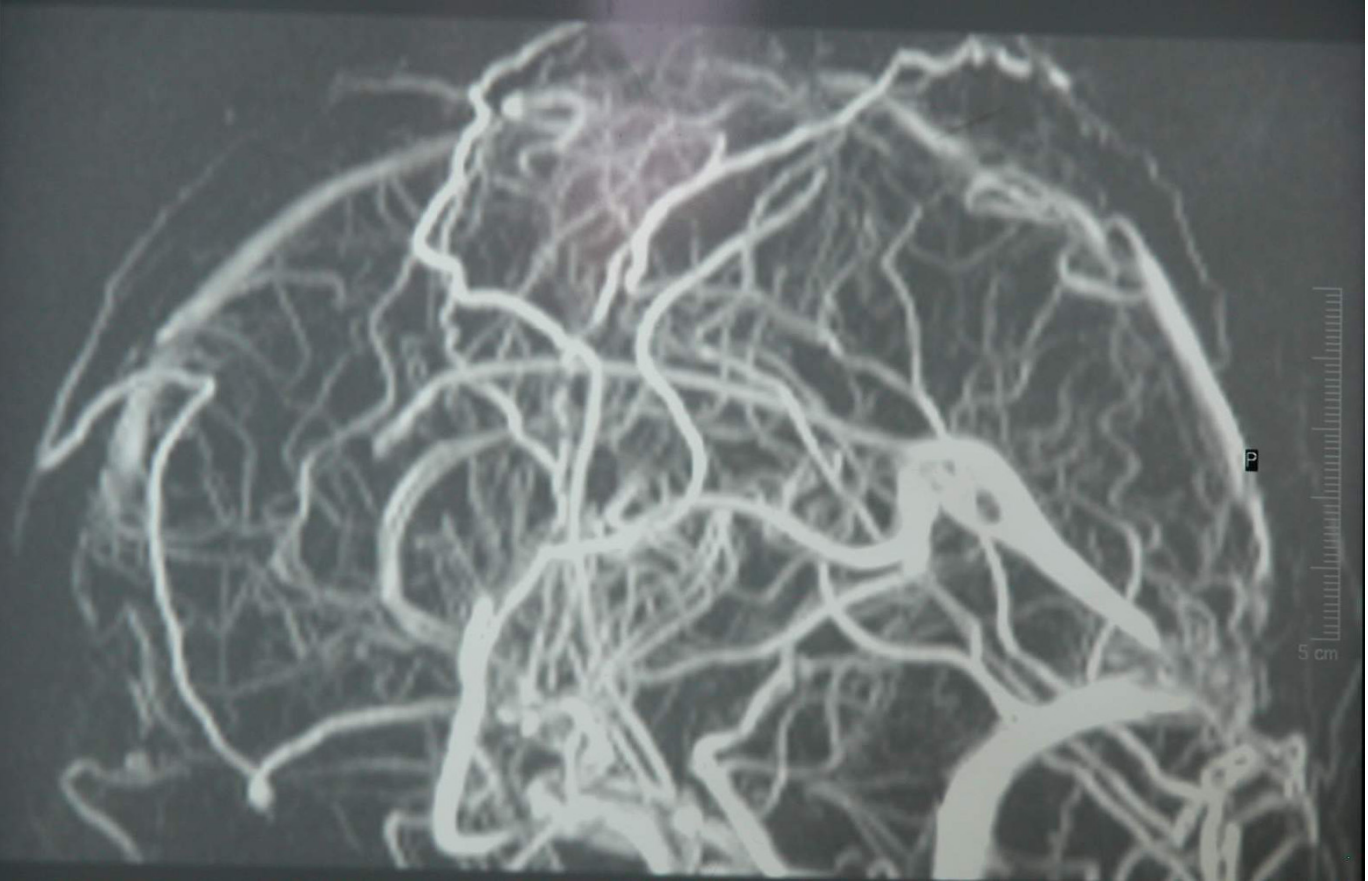
2D MR venogram shows sagital sinus thrombosis.

## Pre-publication history

The pre-publication history for this paper can be accessed here:


